# Total Infectome Characterization of Respiratory Infections during the 2022–23 COVID-19 Outbreak in China Revealed Extensive Coinfections with Links to SARS-CoV-2 Status, Age, and Disease Severity

**DOI:** 10.3390/pathogens13030216

**Published:** 2024-02-29

**Authors:** Zhongzhou Yang, Kanru Cai, Yuqi Liao, Wei-Chen Wu, Li Xing, Minxuan Hu, Jiali Ren, Jieyun Zhang, Xiuyun Zhu, Ke Yuan, Shunyao Wang, Hui Huang, Chunhui Yang, Mingxia Zhang, Mang Shi, Hongzhou Lu

**Affiliations:** 1 Shenzhen Key Laboratory of Systems Medicine for Inflammatory Diseases, School of Medicine, Shenzhen Campus of Sun Yat-sen University, Sun Yat-sen University, Shenzhen 518107, China; yangzhzh26@mail.sysu.edu.cn (Z.Y.); liaoyq23@mail2.sysu.edu.cn (Y.L.); wuweixiongde@126.com (W.-C.W.); humx8@mail2.sysu.edu.cn (M.H.); yangchh33@mail2.sysu.edu.cn (C.Y.); 2 Institute for Hepatology, National Clinical Research Center for Infectious Disease, The Third People’s Hospital of Shenzhen, Shenzhen 518112, China; dabi1982@outlook.com (K.C.); yunyun3152@163.com (J.Z.); sowin@163.com (X.Z.); zmxmby@outlook.com (M.Z.); 3 BGI Genomics, BGI-Shenzhen, Shenzhen 518000, China; xingli@bgi.com (L.X.); renjiali@bgi.com (J.R.); yuanke@bgi.com (K.Y.); wangshunyao@bgi.com (S.W.); huanghui@bgi.com (H.H.); 4 Department of Infectious Diseases, National Clinical Research Center for Infectious Diseases, The Third People’s Hospital of Shenzhen, Shenzhen 518112, China

**Keywords:** total infectome, coinfection, COVID-19, respiratory infection

## Abstract

Between 7 December 2022 and 28 February 2023, China experienced a new wave of COVID-19 that swept across the entire country and resulted in an increasing amount of respiratory infections and hospitalizations. The purpose of this study is to reveal the intensity and composition of coinfecting microbial agents. In total, 196 inpatients were recruited from The Third People’s Hospital of Shenzhen, and 169 respiratory and 73 blood samples were collected for metagenomic next-generation sequencing. The total “Infectome” was characterized and compared across different groups defined by the SARS-CoV-2 detection status, age groups, and severity of disease. Our results revealed a total of 22 species of pathogenic microbes (4 viruses, 13 bacteria, and 5 fungi), and more were discovered in the respiratory tract than in blood. The diversity of the total infectome was highly distinguished between respiratory and blood samples, and it was generally higher in patients that were SARS-CoV-2-positive, older in age, and with more severe disease. At the individual pathogen level, HSV-1 seemed to be the major contributor to these differences observed in the overall comparisons. Collectively, this study reveals the highly complex respiratory infectome and high-intensity coinfection in patients admitted to the hospital during the period of the 2023 COVID-19 pandemic in China.

## 1. Introduction

In early December 2022, following the relief of COVID-19 restrictions in China, the disease quickly swept through the population across the nation, which was followed by a rapid increase in hospitalization due to respiratory illness [1]. Although most clinical testing data remain unclear, it is suspected that the majority of the respiratory cases admitted to hospitals at that time resulted from SARS-CoV-2 infection [2]. Some respiratory diseases are directly associated with SARS-CoV-2, whereas others are caused by secondary infection, coinfections, or comorbidities [3], making it a unique time to study COVID-19-related disease manifestations. 

Coinfections have been commonly reported with the involvement of the RNA virus [4,5,6], DNA virus [7,8,9], bacteria [10,11,12], and fungi [13,14,15] in COVID-19 patients, although the coinfection rates varied from 3.6 to 42.8% from a meta-analysis study [3,10,16]. In more severe cases, SARS-CoV-2 causes dysbiosis in the respiratory tract, which results in the prevalence of other pathogens or opportunistic pathogens in the respiratory tract, and some of these pathogens might be associated with a severe manifestation of the disease or even death [4]. In China, one study focused on bacterial coinfection examined 546 COVID-19 patients sampled from December 2022 to January 2023 in Wuhan, and it revealed that 20.18% of cases are coinfected with bacterial pathogens, such as *Haemophilus influenza*, *Staphylococcus aures*, *Pseudomonas aeruginosa*, and *Streptococcus penumonia* [17]. Despite this, it is still unclear what the full spectrum of infectious agents is, namely, viruses, bacteria, and fungus, and their potential association with COVID-19.

Therefore, our research aims to systematically reveal all types of microbial pathogens, namely, “total infectome” for inpatients subject to respiratory infections during the 2022 COVID-19 pandemic in China [18,19,20]. In order to characterize all types of pathogens, we utilized the metagenomic next-generation sequencing (mNGS) approache [21], which is widely used to reveal pathogens involved in lower respiratory tract infection [22], pneumonia [23], or other infectious diseases in the respiratory tract [24,25]. In addition, the diversity of the total infectome, i.e., alpha and beta diversity, was estimated and compared across different groups defined by the SARS-CoV-2 detection status, age groups, and severity of the disease. The impact of these factors, as well as clinical factors, on the prevalence and abundance of each pathogen was also examined.

## 2. Materials and Methods

### 2.1. Sample and Clinical Data Collection

In this study, we collected 169 respiratory and 73 blood samples from 196 inpatients diagnosed with respiratory infections via computed tomography at The Third People’s Hospital of Shenzhen during the period from 8 December 2022 to 20 February 2023. The respiratory samples included 143 bronchoalveolar lavage fluid samples and 22 sputum samples, and the blood samples were collected using EDTA tubes to prevent blood clots. All samples were taken after the patients were admitted to the hospital, and the metagenomic next-generation sequencing of these samples was carried out upon the request of clinicians for in-depth diagnostic purposes. For each of the patients, information such as demographic data, comorbidities, clinical symptoms, treatment, and test results from respiratory and blood samples were collected for later comparisons relative to microbial diversity and abundance.

### 2.2. Sample Processing and DNA Extraction

For respiratory samples, 1.5–3 mL sputum or bronchoalveolar lavage fluid (BALF) samples were collected from patients according to standard procedures. Sputum was pretreated with 0.1% DTT (dithiothreitol) for 30 min at room temperature to liquefy the sample. Then, the samples were fully mixed with 55 µL of NaCl (5 M), 10 µL of MgCl_2_ (100 mM), and 10 µL of HL-SAN and incubated at 30 °C for 10 min for the dehosting process. Next, a wall-lysing enzyme was added prior to the addition of microglass grinding beads (MGI Tech Co., Ltd., Wuhan, China), and the samples were vortexed at 1000× *g* for 20 min. Supernatants were subsequently collected and subjected to DNA extraction. For blood samples, 3 mL of blood samples was taken from patients, followed by centrifugation at 1600× *g* for 10 min at 4 °C within 8 h of collection for plasma separation. Plasma samples were transferred to a new sterile tube. For both BALF and blood samples, the DNA of all samples was extracted using the TIANamp Micro DNA Kit (DP316, Tiangen Biotech, Beijing, China) following the manufacturer’s operational manual. 

### 2.3. Metagenomic Sequencing

The extracted DNA was subsequently used for the construction of DNA libraries, which were constructed via DNA fragmentation, end repair, adapter ligation, and PCR amplification using the DNA construction kit (MGI Tech Co., Ltd., Wuhan, China) [26]. The fragment size of the quality control library was about 250 to 350 bp using the Agilent 2100 Bioanalyzer (Agilent Technologies, Santa Clara, CA, USA). DNA nanoballs (DNBs) were subsequently prepared from the library using rolling circle amplification. Next, the qualified libraries were prepared and sequenced on the MGI-SEQ 2000 platform, MGI Tech, Shenzhen, China [27].

### 2.4. Bioinformatic Analysis

High-quality sequencing data were generated by filtering out low-quality reads from raw sequencing data. Then, Burrows–Wheeler Alignment was used for mapping to the human reference genome (hg19) to remove human host DNA sequences [28]. After removing low-complexity reads, the remaining data were classified by aligning to the Pathogens Metagenomics Database (PMDB), which consisted of genomes for 10,989 bacteria, 1179 fungi, 5050 viruses, and 282 parasites. All reference genomes in the database were downloaded from the National Center for Biotechnology Information (NCBI). The quantification of each species was estimated based on the reads per million of the total number of reads in the library (i.e., RPM). For each library, microbes were regarded as positive when their RPM > 2.

### 2.5. Evolutionary Analyses

Marker genes were selected, based on coverage and abundance levels, for each microbial species identified in this study, based on which evolutionary analyses were performed. Accordingly, genes of closely related microbial taxonomy were also obtained from the NCBI/GenBank database. These sequences were subsequently aligned using the FFT-NS-I algorithm implemented in MAFFT (version 7.490) [29], followed by the removal of ambiguously aligned regions using the TrimAl program [30]. Phylogenetic analyses were performed for each of the aligned gene sequences using the maximum likelihood algorithm implemented in the program PhyML (version 3.0), using SPR branch swapping and the GTR nucleotide substitution model [31]. The support of each node in the phylogeny was estimated using the approximate likelihood ratio (aLRT) test that is also implemented in PhyML.

### 2.6. Statistical Analyses

The alpha- and beta- microbial diversities for the microbial groups identified in this study were estimated using the vegan package implemented in *R*. Alpha diversities were evaluated and compared using the Simpson index. The Kruskal–Wallis test was used to evaluate the statistical significance of comparisons between two groups. Beta diversity was calculated as the Bray–Curtis dissimilarity between groups, and group comparisons were performed using permutational multivariate analysis of variance (PERMANOVA). Finally, the Pearson correlation coefficient was used to find the correlation between clinical indicators, clinical factors, and abundance levels of each microbial species.

## 3. Results

### 3.1. Participant Recruitment and Clinical Factors

**Figure 1:**
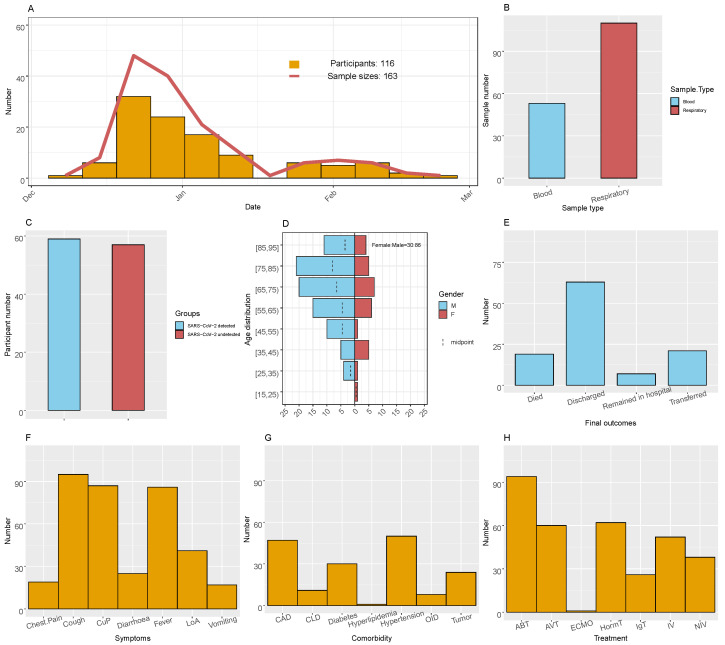
Clinical features of inpatients enrolled in this study. (**A**) Timing of the sampling. (**B**) Frequency of sample types. (**C**) Frequency of patients with detected and undetected SARS-CoV-2 virus. (**D**) Age distribution for male and female inpatients. (**E**) Frequency of final outcomes. (**F**) Frequencies of symptoms. CuP = Cough up phlegm; LoA = loss of appetite. (**G**) Frequencies of comorbidities. CAD = Coronary artery disease; CLD = chronic lung disease; OID = other infectious disease. (**H**) Frequencies of treatment. NIV = Non-invasive ventilation; IV = invasive ventilation; ECMO = extracorporeal membrane oxygenation; AVT = antiviral therapy; ABT = antibiotic therapy; HormT = hormone therapy; IgT = immunoglobulin therapy.

A total of 196 inpatients who developed respiratory infection symptoms and were diagnosed with pulmonary infections via computed tomography were involved in the present study (Table 1). The sampling of these patients was carried out during the period from 7 December 2022, when the Chinese government ended its zero-COVID-19 policy, to 20 February 2023 (Figure 1A). Among these patients, 39 (20%) were excluded from the following analyses because there were no respiratory or blood samples, and 41 (21%) were excluded because they had pre-existing conditions, such as HIV infection, organ transplantation, or cancer. Therefore, the final number of patients involved in this study was 116, from whom respiratory (*n* = 110) and/or blood samples (*n* = 53) were collected for subsequent mNGS testing (Figure 1B and Table S1). In total, 83 (72%) inpatients provided one sample type and 33 inpatients (27%) provided at least one sample type with 2 to 5 samples. Among the enrolled patients, around half (59, 51%) of the patients tested positive for COVID-19 at the hospital (Figure 1C). The rest of the patients tested negative or not tested for the SARS-CoV-2 virus, although a recent history of SARS-CoV-2 infection could not be excluded. The age of these patients ranged from 15 to 95 (median 69), with more males (*n* = 86, 74% of all patients) than females (*n* = 30, 26%) in general (Figure 1D). As for the reported clinical outcomes of these patients (*n* = 116), 19 (16%) resulted in death, with 15 (13%) exhibiting 28-day mortality; 21 (18%) were transferred to other hospitals, whereas the majority (*n* = 63, 54%) were recovered and discharged from the hospital (Figure 1E), with an average of 23 days with respect to staying in the hospital. In total, 49 patients (42%) were admitted to the ICU during hospitalization, amongst which 37 were subject to invasive ventilation. Almost all patients developed common respiratory infections, with the highest rate observed with respect to coughing (*n* = 95, 82%) and coughing up phlegm (*n* = 87, 75%) (Figure 1F). The comorbidities of these patients included hypertension (*n* = 50, 43%), coronary artery disease (CAD, *n* = 47, 41%), and diabetes (*n* = 30, 26%) (Figure 1G). Many patients underwent anti-bacterial (*n* = 94, 81%), anti-viral (*n* = 60, 52%), and hormonal treatment (*n* = 62, 53%), whereas only one had ECMO (*n* = 1, 1%) (Figure 1H).

### 3.2. Total Infectome and Coinfection Characterizations

**Figure 2:**
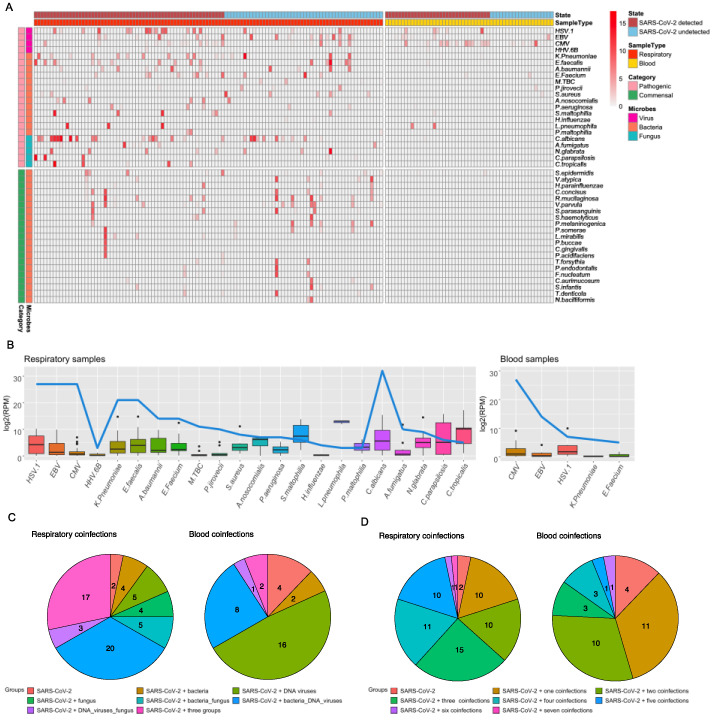
Diversity, prevalence rate, and con-infection frequencies of all types of pathogens within the patients enrolled in this study. (**A**) Heat map indicating the abundance and prevalence of microbes between pathogenic and commensal taxa. The x-axis samples are categorized as SARS-CoV-2-detected and SARS-CoV-2-undetected from four sample types, consisting of respiratory samples and blood samples. The y-axis is grouped as virus, bacteria, and fungus, which were ordered by the number of samples. (**B**) The prevalence of taxa abundance and sample size for the coinfections. HSV-1 = *Human alpha herpesvirus 1*; EBV = *Human gamma herpesvirus 4*; CMV = *Human betaherpesvirus 5*; HHV. 6B = *Human betaherpesvirus 6B*; K. Pneumoniae = *Klebsiella Pneumoniae*; E. faecalis = *Enterococcus faecalis*; A. baumannii = *Acinetobacter baumannii*; E. Faecium = *Enterococcus faecium*; M. TBC = *Mycobacterium tuberculosis complex*; P. jirovecii = *Pneumocystis jirovecii*; S. aureus = *Staphylococcus aureus*; A. nosocomialis = *Acinetobacter nosocomialis*; P. aeruginosa = *Pseudomonas aeruginosa*; S. maltophilia = *Stenotrophomonas maltophilia*; H. influenzae = *Haemophilus influenzae*; L. pneumophila = *Legionella pneumophila*; P. maltophilia = *Pseudomonas maltophilia*; C. albicans = *Candida albicans*; A. fumigatus = *Aspergillus fumigatus*; N. glabrata = *Nakaseomyces glabrata*; C. parapsilosis = *Candida parapsilosis*; C. tropicalis = *Candida tropicalis*. (**C**) Pie charts for the types of coinfection infectome between respiratory and blood samples in all detected SARS-CoV-2 infections. (**D**) Pie charts for the number of coinfections between respiratory and blood samples in all detected SARS-CoV-2 infections.

**Figure 3:**
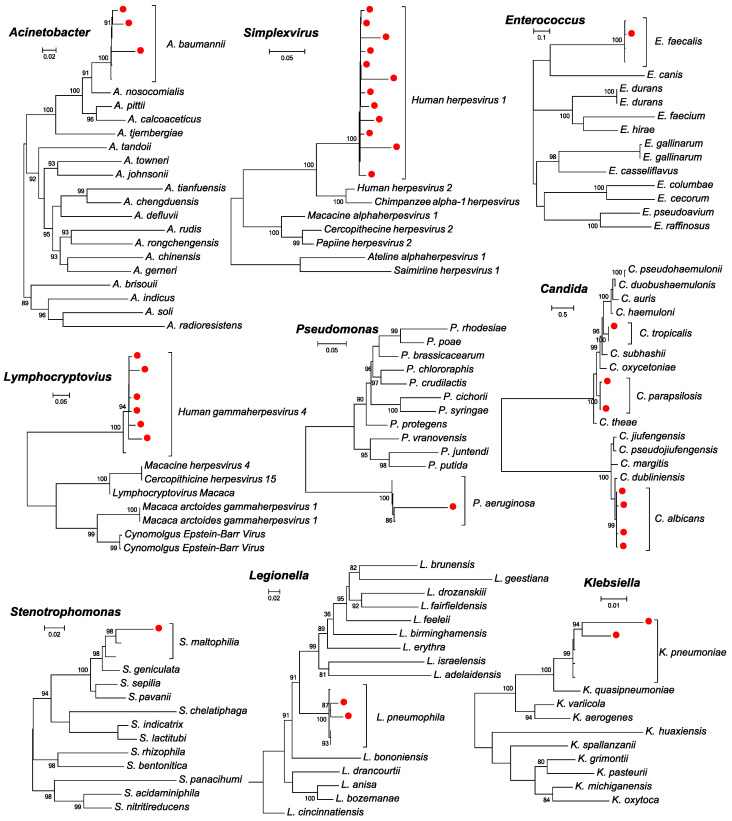
Identification of major pathogens or opportunistic pathogens at the species level. Pathogens identified in this study and those with high abundance levels were analyzed in the context of related microbes from the same genus. The phylogenies were reconstructed based on *rpob*, *gyrb*, *rpb2*, *ebna1*, and *ul30* genes and using the PhyML program. The pathogens identified from this study are marked by a red solid circle. The corresponding names of the species and genus were marked to the right of the tree.

Following the DNA metagenomic sequencing of 163 samples from 116 inpatients, a total of 22 species of coinfecting microbial pathogens (Table S2) and 21 commensal microbes (Table S3) were detected in this study, among which 22 pathogens were detected in respiratory samples and 5 pathogens were detected in blood (Figure 2). Species identification was confirmed with phylogenetic analyses based on the marker genes of the corresponding virus, bacteria, and fungi species (Figure 3). The pathogens detected included 4 species of virus, 13 species of bacteria, and 5 species of fungus, most of which were opportunistic pathogens, whereas the commensal microbes were all bacterial species, including *Prevotella pleuritidis*, *Streptococcus parasanguinis*, and *Prevotella melaninogenica* (Figure 2A). In the respiratory samples, *Candida albicans* (29%), HSV-1 (25%), EBV (25%), and CMV (25%) were among the most prevalent microbes, whereas *Legionella pneumophila* (6799 RPM), *Candida tropicalis* (416 RPM), and *Stenotrophomonas maltophilia* (256 RPM) had the highest median abundance levels estimated based on all samples (Figure 2B). In the blood, CMV (46%) and EBV (24%) were the most commonly identified pathogens, although at much lower abundance levels (8 and 4 RPM, respectively) (Figure 2B).

We next described the coinfection rates and patterns across 60 respiratory and 33 blood samples associated with patients who were SARS-CoV-2-positive at the hospital. Among the samples examined here, coinfections with different types of pathogens were the norm rather than exceptions (Figure 2C). In the 60 SARS-CoV-2 respiratory samples detected, the most common scenario was bacterial and DNA virus coinfections (33%), and coinfections with all three types of pathogens (DNA virus, bacteria, and fungi) were also frequently detected (28%) (Figure 2C). In the blood, however, DNA virus coinfection was more frequently detected (48%). Furthermore, 38% of the respiratory samples and 15% of the blood samples examined here were coinfected with more than three pathogen species, with the highest coinfection rates reaching seven species in total (Figure 2D).

### 3.3. Comparisons of Diversities across Different Sample Types, SARS-CoV-2 Status, Age, and Disease Severity

**Figure 4:**
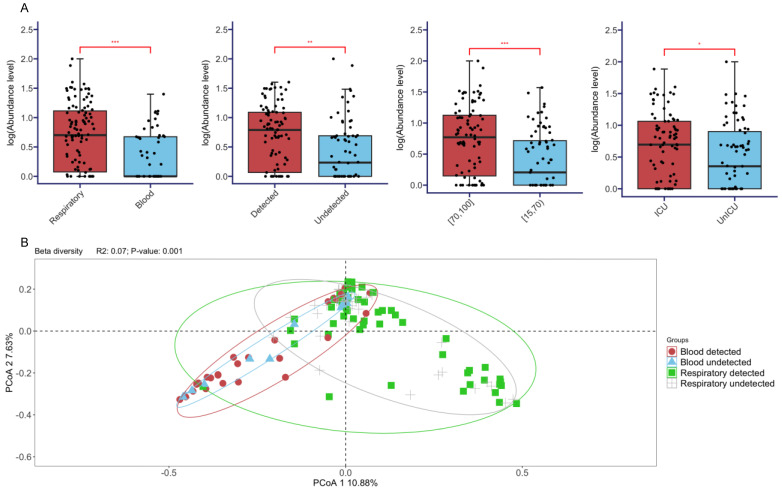
Comparisons of overall diversities of pathogens. (**A**) Alpha diversities were compared across different sample types (respiratory tract versus blood), SARS-CoV-2 status (detected versus undetected), age groups (15 to 70 versus 70 to 100), and severity (ICU versus non-ICU). The statistical significance of the comparisons (Wilcoxon test) is marked on top of the boxplot: * (*p* < 0.05); ** (*p* < 0.01); *** (*p* < 0.001). (**B**) Beta diversity was compared to the microbial compositions between respiratory and blood through the PCoA. The significant test of comparisons (PERMANOVA test) is annotated in the top-left position.

The overall diversity of pathogens, measured by the Shannon index, was compared across different groups (Figure 4A). Generally, significantly higher diversities were observed in the respiratory tract (*p* < 0.001, Kruskal–Wallis test), SARS-CoV-2-detected patients (*p* < 0.01, Kruskal–Wallis test), elderly patients (>70 age group, *p* < 0.001, Kruskal–Wallis test), and patients who had more severe disease symptoms, namely, those subject to the ICU (*p* < 0.05, Wilcox test). We further compared the microbial compositions between different groups, which revealed marked differences between the two sample types (*R*^2^ = 0.07, *p* < 0.001 under PERMANOVA test) (Figure 4B). Furthermore, given the same sample type, the microbial compositions were further compared, which revealed significant differences among the SARS-CoV-2 status (*R*^2^ = 0.01; *p* = 0.007), age (*R*^2^ = 0.01; *p* = 0.002), and disease severity (*R*^2^ = 0.01; *p* = 0.006) under the PERMANOVA test.

### 3.4. Association of Coinfecting Pathogens with Detection Status, Age, and Disease Severity

**Figure 5:**
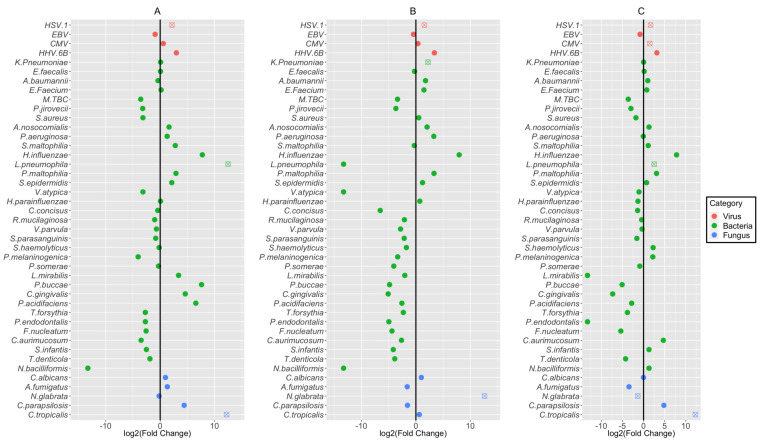
Association of each microbial species with the SARS-CoV-2 status, age, and severity. (**A**) SARS-CoV-2 detection status for 43 coinfecting microbes among the virus (red), bacteria (green), and fungus (blue). The “X” mark in circles denotes a significant difference. (**B**) Age of impact between 15 and 70 and elderly patients (i.e., 70–95 age groups) for 43 coinfecting microbes, with the same mark in (**A**). (**C**) Disease severity (i.e., whether the patient stayed in the ICU) for 43 coinfecting microbes, with the same mark in (**A**).

We next compared the fold changes of microbial species that corresponded to the groups defined by the SARS-CoV-2 detection status, age, or disease severity (i.e., whether the patient stayed in the ICU) (Figure 5). These comparisons were performed based on 43 coinfecting microbes, which included 4 species of viruses, 34 species of bacteria (i.e., 13 pathogenic and 21 commensal bacteria), and 5 species of fungus, after adjusting for all clinical covariates (listed in Table S1) via the IPTW propensity score. At *p* < 0.05 levels, HSV-1, *L. Pneumoniae*, and *C. tropicalis* were more enriched in patients who were positive for SARS-CoV-2; three pathogenic microbes, HSV-1, *K. Pneumoniae*, and *N. glabrata*, were more enriched in elderly patients (i.e., 70–95 age groups); four pathogens (HSV-1, CMV, *L. Pneumoniae*, and *C. tropicalis*) were more enriched in patients who stayed in the ICU, whereas the fungus *N. glabrata* was more enriched in patients who did not stay in the ICU.

### 3.5. Correlations of Clinical Indicators and Pathogen Abundance

**Figure 6:**
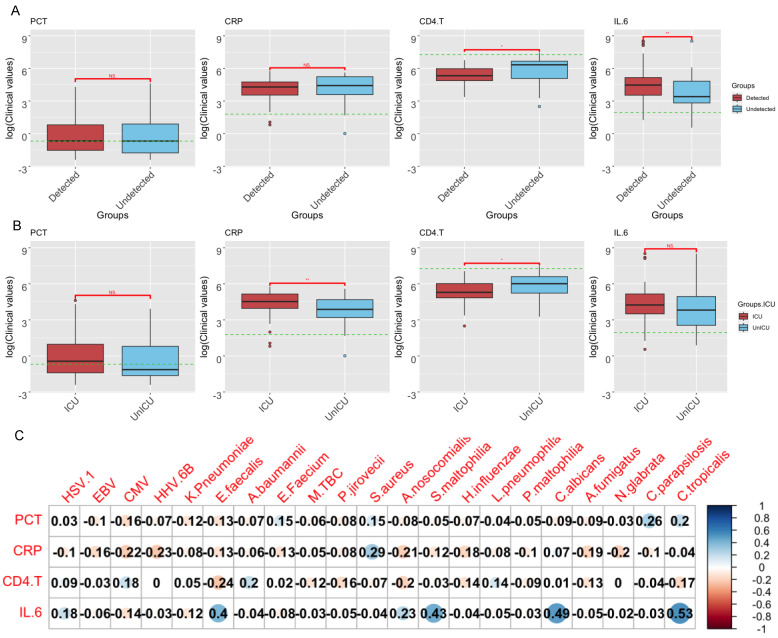
Association between clinical factors and the abundance levels of pathogenic microbes. (**A**) The correlation between four clinical indicators and the SARS-CoV-2 detection status. The green dashed lines represent the threshold for each clinical indicator. The statistical significance of comparisons (Wilcoxon test) is marked on top of the boxplot: * (*p* < 0.05); ** (*p* < 0.01). (**B**) The correlation between four clinical indicators and disease severity (i.e., whether the patient stayed in the ICU), with the same threshold line and statistically significant differences in (**A**). (**C**) Correlation heatmap between four clinical indicators and pathogenic microbiomes by the order of virus, bacteria, and fungus.

To reveal the interactions between microbes and host responses, we studied the correlations of microbial abundance with four clinical indicators in the blood, namely, procalcitonin (PCT), C-reactive protein (CRP), CD4.T, and IL.6 (Figure 6A). We first examined the relationship between clinical factors and the SARS-CoV-2 detection status or disease severity, which revealed that increasing levels of CD4.T were associated with both the SARS-CoV-2-positive group (*p* < 0.001, Wilcox test) and those with more severe disease (*p* < 0.001, Wilcox test), whereas increasing levels of IL.6 were associated with the SARS-CoV-2-positive group (*p* < 0.001, Wilcox test). We then compared the abundance levels of each microbial species with each of the clinical indicators, which indicated a strong positive correlation between IL6 and fungal species *Candida tropicalis* and *Candida albicans* (Figure 6B), although no obvious associations were observed between clinical factors and commensal microbes.

## 4. Discussion

Our study reveals high frequencies of viral, bacterial, and fungal pathogens from patients who were experiencing respiratory illness during the period of a major COVID-19 outbreak in China. Other studies have also investigated coinfecting bacteria and viruses during the same period in Wuhan, which suggested *Haemophilus influenza*, *Staphylococcus aureus*, and *Streptococcus pneumonia* as the most prevalent coinfecting microbes [17]. Nevertheless, our study, which was based at a hospital in Shenzhen, identified much less of the above-mentioned microbes and instead revealed *Klebsiella Pneumoniae*, *Enterococcus faecalis*, and *Acinetobacter baumannii* as the most prevalent bacterial pathogens in SARS-CoV-2-positive patients. Furthermore, highly prevalent DNA viruses identified here, such as HSV and EBV, were not identified in the Wuhan study [17], although they were frequently identified in severe COVID-19 patients from previous studies [4,5,21]. Therefore, there are major differences in dominant species despite the fact that the samples were obtained from the same outbreak. One possibility for such striking differences is that the patient groups of these two studies were from different hospitals and regions, which might harbor distinctive pathogens or opportunistic pathogens. Alternatively, since the study based in Wuhan used medical records as evidence for microbial detection, it is unclear which samples (upper or lower respiratory tract) or what approaches were used to identify the dominant microbial species. Indeed, the spectrum of microbial diversity differs significantly between upper or lower respiratory tract samples [5,32], which should not be overlooked when comparing microbial diversity and abundance. Finally, an important factor that affects the microbial composition of respiratory samples may be antimicrobial treatment, and it has been reported that antimicrobial treatment is more intense in the earlier phase of the COVID-19 outbreak than in the later phase [33]. Therefore, this could be a source of variation with respect to the composition of the infectome.

In addition to bacteria, our results also revealed that fungal and viral DNA pathogens might play important roles in more severe disease outcomes. Indeed, these pathogens might play an important role in the disease manifestation of the respiratory tract and blood, followed by primary infection, and therefore should be taken into account when studying coinfection and super-infection [34,35]. For example, herpesvirus coinfection, particularly with HSV-1, is thought to be associated with severe respiratory infections [36]. Herpesvirus coinfection may cause orolabial herpes to reactivate and progress into pulmonary or tracheal infection, resulting in infiltrated inflammatory cells in lung necrosis, alveolar hemorrhage, and parenchyma and leading to severe disease [5,37,38]. Fungal coinfections are relatively less pathogenic, but in some cases, for example, Aspergillus coinfection during the SARS-CoV-2 delta variant outbreaks, it can also result in high morbidity and mortality [39,40]. In our study, the most prevalent and relevant fungi identified were *Candida* spp., and they were mainly discovered in the respiratory but not in blood samples. Interestingly, our results indicated that the increasing level of IL-6 positively correlated with the abundance level of *Candida albicans* and *Candida tropicalis*.

Our study revealed significant differences in microbial composition and diversity between the SARS-CoV-2-detected and -undetected groups. Despite the overall differences, the beta diversity showed substantial overlap in the samples from these two groups, suggesting similarities between the two groups. Furthermore, at individual pathogen levels, only a few showed significant differences, while other pathogens had similar abundance levels between the two groups, including the most dominant pathogens *C. albicans* and HSV-1. It is possible that even though some of the cases are marked as SARS-CoV-2-undetected, these might be related to SARS-CoV-2 infection. It is undetected because SARS-CoV-2 loads decrease with the progression of infection even though patients remain sick [41]. Alternatively, the pathogen profiles for secondary respiratory and opportunistic infection are similar within the same hospital regardless of what the primary pathogen is.

Our study has several limitations. First, while the COVID-19 outbreak is widespread across China, our investigation was carried out at a single hospital in a single city. Although the hospital is dedicated to COVID-19 patients in Shenzhen, it has limited power in presenting the coinfection status for the entire outbreak. Second, the method used here is only based on DNA metagenomic sequencing, and as a result, coinfecting RNA pathogens could not be revealed from our data. Therefore, the full spectrum of pathogen diversity and the coinfection landscape remains to be examined via multi-centered studies that are more comprehensive, and multi-omics total infectome approaches will probably be implemented.

## 5. Conclusions

Our study provides a systematic and cross-sectional investigation of the respiratory microbial pathogens present in a hospital during a major COVID-19 outbreak. Although small in scale, it reveals one of the highest coinfection rates so far and underlines the importance and complexity of the infectome associated with COVID-19-related disease manifestations. Future research is needed to reveal how the infectome interacted with SARS-CoV-2 patients and how interactions resulted in more severe disease outcomes.

## Figures and Tables

**Table 1:** Characteristics of patients.

Patient Characteristics	SARS-CoV-2 Undetected (*n* = 57)	SARS-CoV-2 Detected (*n* = 59)
Age	60.2 (15~91)	70.7 (31~91)
Gender [Number (%)] Male Female	38 (66.7) 19 (33.3)	48 (81.4) 11 (18.6)
Outcomes [Number (%)] Died Discharged Transferred Undischarged NA	5 (8.8) 36 (63.1) 8 (14.0) 3 (5.3) 5 (8.8)	14 (23.7) 27 (45.8) 13 (22.0) 4 (6.8) 1 (1.7)
Severity [Number (%)] ICU UnICU	17 (29.8) 40 (70.2)	32 (54.2) 27 (45.8)
Symptoms [Number (%)] Chest.Pain Cough Cough up Phlegm (CuP) Diarrhoea Fever Loss of Appetite (LoA) Vomiting	9 (15.8) 42 (73.7) 36 (63.2) 7 (12.3) 38 (66.7) 15 (26.3) 8 (14.0)	10 (16.9) 53 (89.8) 51 (86.4) 18 (30.5) 48 (81.4) 26 (44.1) 9 (15.3)
Comorbidities [Number (%)] Coronary Artery Disease (CAD) Chronic Lung Disease (CLD) Diabetes Hyperlipidemia Hypertension Other infectious disease (OID) Tumor	16 (28.1) 4 (7.0) 14 (24.6) 0 (0) 18 (31.6) 4 (7.0) 10 (17.5)	31 (52.5) 7 (11.9) 16 (21.7) 1 (1.7) 32 (54.2) 4 (6.8) 14 (23.7)
Treatment [Number (%)] Antibiotic therapy (ABT) Antiviral therapy (AVT) ECMO Hormone therapy (HormT) Immunoglobulin therapy (IgT) Invasive ventilation (IV) Non-Invasive ventilation (NIV)	44 (77.2) 11 (19.3) 1 (1.75) 20 (35.09) 8 (14.04) 13 (22.81) 15 (26.32)	50 (84.7) 49 (83.1) 0 (0) 42 (71.2) 18 (30.5) 39 (66.1) 23 (40.0)

## Data Availability

The metagenomic sequencing reads (non-host, non-ribosomal RNA reads) from 163 clinical specimens generated in this study were deposited in the CNSA (CNGB Sequence Archive) of CNGBdb (China National GeneBank DataBase, https://db.cngb.org/cnsa/, accessed on 19 February 2024; project accession: CNP0004840).

## Supplementary Materials

The following supporting information can be downloaded at https://www.mdpi.com/article/10.3390/pathogens13030216/s1. Table S1: Details of clinical data for the final 116 inpatients; Table S2: Details of 91 pathogenic microbes identified in this study; Table S3: Details of 211 commensal microbes identified in this study.
